# Gastric ultrasound to assess the prokinetic efficacy of erythromycin in a patient taking glucagon‐like peptide‐1 receptor agonists

**DOI:** 10.1002/anr3.70008

**Published:** 2025-04-15

**Authors:** N. S. Sidhu

**Affiliations:** ^1^ Department of Anaesthesia, School of Medicine University of Auckland Auckland New Zealand; ^2^ Department of Anaesthesia and Perioperative Medicine North Shore Hospital Auckland New Zealand

**Keywords:** gastric ultrasound, glucagon‐like peptide‐1 receptor agonist, prokinetic agents

## Abstract

Glucagon‐like peptide‐1 receptor agonists slow gastric emptying and may increase aspiration risk. Recent guidelines suggest using prokinetic agents pre‐operatively, but no studies have assessed the efficacy of erythromycin for this purpose. We present a 53‐year‐old man (weight 110 kg) taking liraglutide and undergoing elective knee arthroscopy. Despite 19 h of fasting and withholding liraglutide, gastric ultrasound revealed a grade 3 antrum with solid content. Intravenous erythromycin 300 mg was administered, causing transient gastrointestinal symptoms. A repeat ultrasound 15 min later showed reduced solid content, although the antrum was not convincingly empty. As the patient declined neuraxial anaesthesia without sedation, a modified rapid sequence induction was performed. An ultrasound scan at the completion of surgery confirmed an empty stomach, and recovery was uneventful. This is the first documented case using gastric ultrasound to assess the effect of erythromycin on a patient taking a glucagon‐like peptide‐1 receptor agonist. While erythromycin achieved its desired effect within 100 min, the optimal timing for prokinetic administration and subsequent ultrasound assessment remains uncertain. Gastric ultrasound may refine risk stratification and guide prokinetic use for these patients. Further research is needed to determine optimal erythromycin dosing, time to desired effect and side effects to optimise peri‐operative management.

## Introduction

Glucagon‐like peptide‐1 receptor agonists (GLP‐1 RAs) are used to manage diabetes and obesity, primarily by reducing gastric emptying rate and increasing satiety. Examples include semaglutide (Ozempic^®^ and Wegovy^®^), dulaglutide (Trulicity^®^), liragluide (Victoza^®^ and Saxenda^®^) and tirzepatide (Mounjaro^®^). In recent years, there has been a rise in their popularity. However, concerns have arisen regarding their potential to increase the risk of pulmonary aspiration by delaying gastric emptying through inhibition of gastric peristalsis and increased pyloric tone. The pooled incidence of residual gastric content in fasted non‐endoscopy patients taking GLP‐1 RAs is estimated at 50% [1]. Recent multidisciplinary guidelines from the United Kingdom [2] and Australasia [3] recommend continuing these drugs in the peri‐operative setting while considering the use of a prokinetic agent. An experimental study in healthy volunteers found that intravenous erythromycin, a macrolide antibiotic, counteracts the glucagon‐like peptide‐1‐induced gastric emptying delay [4]. However, no published studies or case reports have described the use of erythromycin in this patient population.

## Report

A clinical case is presented demonstrating the use of gastric ultrasound to assess the efficacy of erythromycin in a patient taking liraglutide. A 53‐year‐old man weighing 110 kg presented for elective knee arthroscopy with partial medial meniscectomy. His medical history included coronary artery disease (treated with a stent), hypertension, obstructive sleep apnoea, dyslipidaemia, hypothyroidism, mild heartburn and impaired glucose tolerance. His medications included aspirin, rosuvastatin, ezetimibe, losartan, doxazosin, levothyroxine and omeprazole. Five weeks prior to surgery, he commenced liraglutide for weight loss, taking 3 mg daily via subcutaneous injection with his last dose 24 h before surgery. His last meal, consumed 19 h pre‐operatively, consisted of a hamburger, potato chips and two pizza slices, followed by two sweets 13 h prior to surgery. On the morning of surgery, he took 1 g of paracetamol with a sip of water at 07:20.

**Figure 1:**
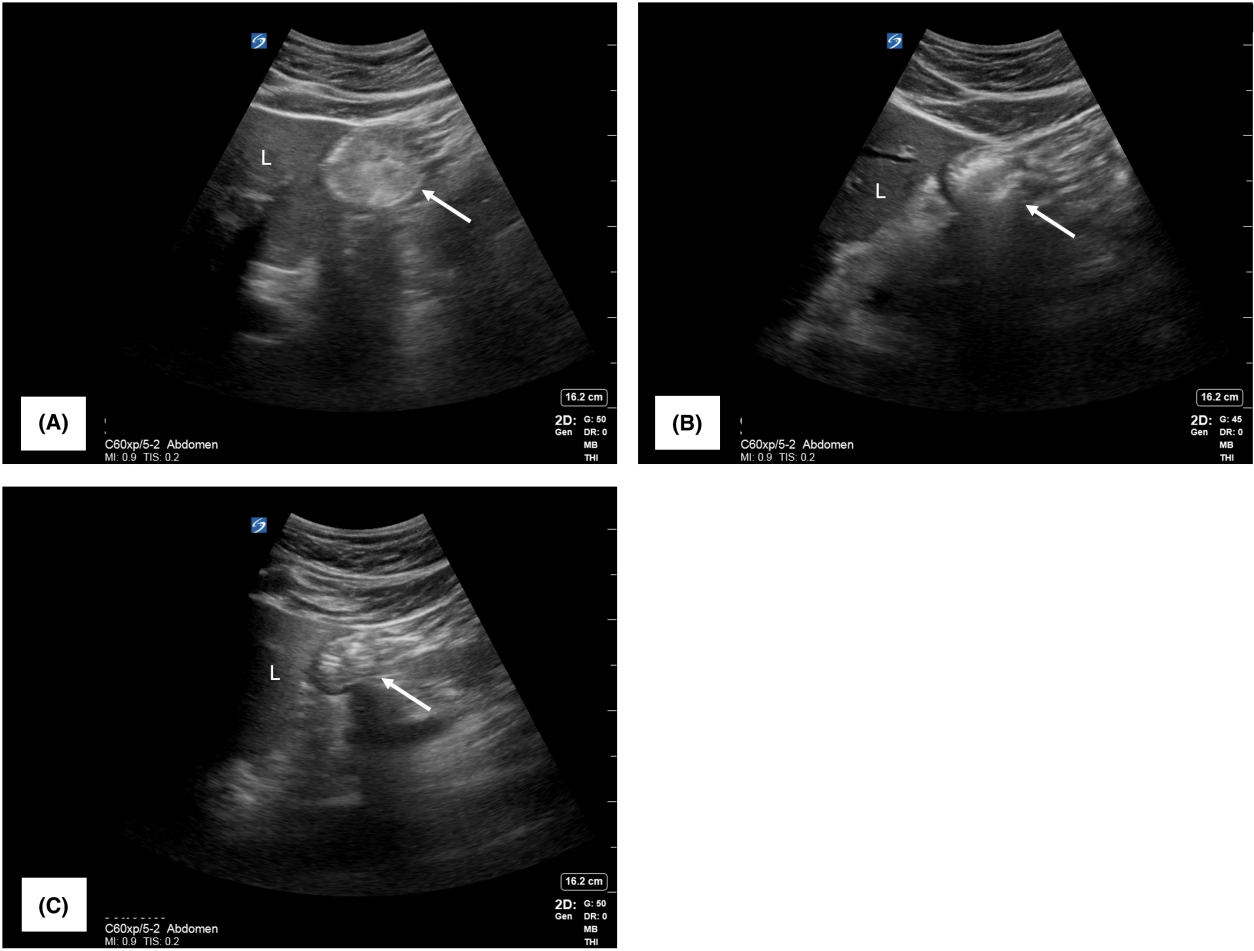
Effect of intravenous erythromycin on gastric emptying in a fasted patient taking GLP‐1 RA assessed using gastric point‐of‐care ultrasound. All images obtained in right lateral decubitus position using a Sonosite™ PX ultrasound machine with a 5‐1 MHz curvilinear transducer (Sonosite, Bothell, Washington, US) on ‘abdominal’ setting at identical depth. (A) Distended gastric antrum with solid content (grade 3). (B) Fifteen minutes after administration of intravenous erythromycin 300 mg, showing a less distended antrum. (C) One hundred minutes after erythromycin administration, gastric antrum is empty (grade 0). GLP‐1 RA, glucagon‐like peptide‐1 receptor agonist; L, liver.

Gastric ultrasound was performed using a Sonosite™ PX with 5‐1 MHz curvilinear transducer (Sonosite, Bothell, Washington, US) as part of the pre‐operative assessment because the patient was on liraglutide, revealing a grade 3 antrum with solid content at 07:50 (Fig. 1A). At 08:00, intravenous erythromycin 300 mg was administered, causing stomach cramps, burping, nausea and sweating within 1 min, lasting approximately 5 min. A repeat scan at 08:15 showed a marked reduction in solid content, although the antrum was not convincingly empty (Fig. 1B). As the patient declined neuraxial anaesthesia without sedation, we proceeded with a modified rapid sequence induction and general anaesthesia. Induction drugs were fentanyl 200 μg, propofol target‐controlled infusion (Marsh model, target Cp 5 μg.ml^−1^, later titrated down to 3 μg.ml^−1^) and rocuronium 100 mg. Bag‐mask ventilation was avoided, cricoid pressure was applied and the trachea was intubated with a size 8 tracheal tube using a Macintosh size 4 videolaryngoscope. A repeat gastric ultrasound at 09:40, at the end of surgery, confirmed an empty antrum (Fig. 1C). Reversal of neuromuscular blockade was performed with sugammadex 200 mg. The patient emerged from anaesthesia uneventfully.

## Discussion

This case describes the use of erythromycin to facilitate gastric emptying in a patient taking GLP‐1 RA with retained gastric content, using gastric ultrasound to assess its efficacy. A joint consensus statement from the Association of Anaesthetists and a number of United Kingdom‐based organisations suggests the use of a prokinetic without specifying a particular agent [2], while an Australasian clinical practice recommendation includes the use of a single intravenous dose of erythromycin at 3 mg.kg^−1^ (up to 250 mg), and waiting 90 to 120 min when possible between administration and induction of anaesthesia, while stating it “*has been shown to accelerate gastric emptying markedly within 15 min*” [3]. While both publications propose gastric ultrasound for initial assessment and risk stratification, UK guidelines do not describe its use to confirm prokinetic efficacy [2] and Australasian guidelines state its use “*may be considered prudent*” but “*there is currently no evidence to date to require this*” [3]. Gastric ultrasound is a highly sensitive and specific diagnostic tool for assessing gastric content with the potential to aid airway management in various settings [5].

The Association of Anaesthetists guideline advises continuing GLP‐1 RA administration throughout the peri‐operative period [2], with the latest Australasian guidelines suggesting the same and to consider withholding once‐daily liraglutide for 3–4 days [3]. This patient omitted liraglutide on the day of surgery, which was the Australasian recommendation at the time. In elective cases, planning allows for assessment, intervention and detailed discussion with the patient. Emergency cases may not allow for the same considerations. While emergency patients are often presumed to have a full stomach and are managed as such, they also have the highest rate of pulmonary aspiration (1:900) [6], warranting careful peri‐operative management.

Our patient was identified as a GLP‐1 RA user ahead of the day of surgery and scheduled second on the operating list with an early arrival in anticipation of performing gastric ultrasound and administering a prokinetic, if necessary. However, due to scheduling changes made without prior notification of the anaesthetist or the surgeon, he was moved to the first slot with staggered arrival times for other patients. Delaying him for up to 2 h for further scanning would have likely resulted in a patient being cancelled from the list. At the time, it was not known to us how long it would take for complete gastric emptying to occur, if at all. The decision to proceed was made in consultation with the surgeon and patient, after outlining the potential risks and benefits, including the option of cancelling surgery and returning some weeks later after stopping liraglutide. A repeat gastric ultrasound scan at the end of the case allowed for aspiration risk stratification during emergence, with the patient now considered low risk for aspiration. Although erythromycin was effective within 100 min in this case, the optimal timing to achieve the desired effect remains uncertain and may be shorter.

Combining the prokinetic action of erythromycin with gastric ultrasound offers promise for refining the peri‐operative management of GLP‐1 RA users. Gastric ultrasound can be used to aid risk assessment and assess prokinetic drug efficacy in patients with delayed gastric emptying. Other prokinetics available in our institution include metoclopramide and domperidone, but neither has been shown to be effective in counteracting the effects of the GLP‐1 peptide hormone [4]. Erythromycin acts as a motilin receptor agonist, stimulating enteric nerves and smooth muscle, triggering the migrating motor complex [7]. Doses of 200–350 mg elicit antral contractions which do not migrate to the small intestine [7]. Future studies should investigate efficacy, optimal dosage and scanning intervals. In the absence of controlled trials, it may be reasonable to scan at 20–30 min intervals after erythromycin administration until the stomach contents are deemed low‐risk for aspiration [5] or no further improvement is observed. While abdominal cramps, nausea and sweating are known side effects of erythromycin at standard doses [8, 9, 10], data on the effect of lower doses are lacking. Our patient experienced transient side effects, as described above.

In conclusion, gastric ultrasound can be a valuable tool for assessing prokinetic efficacy in patients taking GLP‐1 RA with retained gastric content. Incorporating this approach into peri‐operative GLP‐1 RA management guidelines could enhance patient safety. Early identification, along with adequate theatre scheduling and staffing, may improve feasibility. Further research is needed to determine the optimal erythromycin dosing regimen, time to effect, side effect prevalence and severity and differential effects across various GLP‐1 RAs and dosages.
